# Tissue Microarray Analyses Suggest Axl as a Predictive Biomarker in HPV-Negative Head and Neck Cancer

**DOI:** 10.3390/cancers14071829

**Published:** 2022-04-05

**Authors:** Chia-Jung Busch, Christian Hagel, Benjamin Becker, Agnes Oetting, Nikolaus Möckelmann, Conrad Droste, Christina Möller-Koop, Melanie Witt, Markus Blaurock, Sonja Loges, Kai Rothkamm, Christian Betz, Adrian Münscher, Till S. Clauditz, Thorsten Rieckmann

**Affiliations:** 1 Department of Otorhinolaryngology, University Medical Center Hamburg-Eppendorf, 20251 Hamburg, Germany; b.becker@uke.de (B.B.); a.oetting@uke.de (A.O.); c.betz@uke.de (C.B.); n.moeckelmann@marienkrankenhaus.org (N.M.); 2 Department of Otorhinolaryngology, University Medical Center Greifswald, 17475 Greifswald, Germany; markus.blaurock@med.uni-greifswald.de; 3 Institute of Neuropathology, University Medical Center Hamburg-Eppendorf, 20251 Hamburg, Germany; hagel@uke.de; 4 Department of Radiotherapy and Radiation Oncology, University Medical Center Hamburg-Eppendorf, 20251 Hamburg, Germany; k.rothkamm@uke.de; 5 Department of Otorhinolaryngology, Marienkrankenhaus Hamburg, 22087 Hamburg, Germany; muenscher.hno@marienkrankenhaus.org; 6 University Cancer Center Hamburg (UCCH), University Medical Center Hamburg-Eppendorf, 20251 Hamburg, Germany; c.droste@uke.de; 7 Institute of Pathology, University Medical Center Hamburg-Eppendorf, 20251 Hamburg, Germany; c.moeller-koop@uke.de (C.M.-K.); me.witt@uke.de (M.W.); t.clauditz@uke.de (T.S.C.); 8 DKFZ-Hector Cancer Institute, University Medical Center Mannheim, 68167 Mannheim, Germany; sonja.loges@medma.uni-heidelberg.de; 9 Division of Personalized Medical Oncology (A420), German Cancer Research Center (DKFZ), 69120 Heidelberg, Germany; 10 Department of Personalized Oncology, University Medical Center Mannheim, Medical Faculty Mannheim, University of Heidelberg, 68167 Mannheim, Germany

**Keywords:** HNSCC, axl, gas6, tissue microarray (TMA), prognosis, biomarker

## Abstract

**Simple Summary:**

Despite many efforts, no predictive biomarkers that could guide clinical decision making and personalized treatment have been established for patients with head and neck squamous cell carcinoma. We propose that high expression of the tyrosine kinase receptor Axl identifies patients as being at enhanced risk for treatment failure under surgery alone and, hence, should be treated by primary or adjuvant radiotherapy.

**Abstract:**

The receptor tyrosine kinase Axl is described to promote migration, metastasis and resistance against molecular targeting, radiotherapy, and chemotherapy in various tumor entities, including head and neck squamous cell carcinoma (HNSCC). Since clinical data on Axl and its ligand Gas6 in HNSCC are sparse, we assessed the association of Axl and Gas6 expression with patient survival in a single center retrospective cohort in a tissue microarray format. Expression was evaluated manually using an established algorithm and correlated with clinicopathological parameters and patient survival. A number of 362 samples yielded interpretable staining, which did not correlate with T- and N-stage. Protein expression levels were not associated with the survival of patients with p16-positive oropharyngeal SCC. In HPV-negative tumors, Axl expression did not impact patients treated with primary or adjuvant radio(chemo)therapy, but was significantly associated with inferior overall and recurrence-free survival in patients treated with surgery alone. Gas6 was a positive predictor of survival in patients whose treatment included radiotherapy. Associations remained significant in multivariable analysis. Our data question a meaningful contribution of the Axl/Gas6 pathway to radio-resistance in HNSCC and instead suggest that strong Axl expression identifies tumors requiring adjuvant radio(chemo)therapy after surgery.

## 1. Introduction

Head and neck squamous cell carcinoma (HNSCC) is the sixth most common cancer worldwide with more than 890,000 new cases and 450,000 deaths in 2018 [1,2]. HNSCC develops in mucosal epithelial cells that line the oral cavity, pharynx, larynx, and nasal passages. Apart from human papillomavirus (HPV) and p16 expression in oropharyngeal tumors (OPSCC) molecular markers for prognosis or therapeutic guidance are lacking. HPV-positivity generally confers a favorable prognosis, at least in part due to enhanced radiation sensitivity, and various studies are currently testing strategies of de-intensified treatment [3,4,5]. The majority of HNSCC, especially locally advanced stages, require multimodal treatment consisting of surgery followed by (chemo)radiation (RT/RCT) or primary RCT [6]. Further molecular markers are urgently needed to guide therapeutic decision making for individual tumors in order to improve cure rates for patients with HPV-negative tumors and safely de-intensify therapy for those with HPV-positive ones.

A possible marker and potential target in this regard is Axl. Axl is a *Tyro-3*, *Axl*, and *Mer* (TAM) family receptor tyrosine kinase (RTK), a transmembrane protein expressed on the surface of some normal tissues, as well as endothelial and cancer cells [7,8]. It regulates cellular processes including cell survival, proliferation, migration, invasion, adhesion, and motility. The most frequently described ligand for Axl is growth arrest-specific protein 6 (Gas6) [7,8,9]. Gas6 is a large protein that binds to the extracellular domains of Axl and induces its dimerization and subsequent kinase activation. Axl activation initiates a number of downstream pathways such as PI3K, MAPK, and PKC [7,8,9]. Also, Axl is linked with metastasis, invasion, and migration in many tumor entities, making it an attractive therapeutic target [7,8,10]. An association of Axl with tumor development, progression, and adverse prognosis has been shown in several malignancies including lung [10,11,12,13], breast [10,14,15,16], colon [17], prostate [18], and ovarian cancer [19], as well as HNSCC [20,21]. Up-regulation and activation of Axl have also been found to be clinically significant features of resistance to targeted therapies, such as EGF receptor and PI3K inhibition [7,9,10,13]. Therefore, additional targeting of Axl by either Axl inhibitors or through the inhibition of AP-1 transcription factors, c-JUN and c-FOS, which drive Axl expression, represent attractive potential therapeutic options [22,23]. Furthermore, Axl is a driver for epithelial-to-mesenchymal transition (EMT), enabling cells to retain a mesenchymal phenotype enhancing migration and invasion [23,24,25].

In HNSCC, recent publications have described Axl-mediated resistance, not just against the inhibition of other central kinases [22,26,27,28,29], but also against ionizing radiation [20,27,29]. Brand et. al. analyzed the role of Axl in HNSCC cell lines that were reliant on Axl for cellular proliferation, migration, and invasion [20]. Axl protein expression was shown to increase during tumor progression with the highest levels in recurrent tumors [25]. Radiation-resistant HNSCC cell lines and xenografts expressed elevated levels of Axl, indicating a role of Axl as a radio-resistance factor [20,25,27,29]. Small-molecule inhibitors targeting Axl render HNSCC cell lines more sensitive towards radiation, chemotherapy, cetuximab, and PARP-inhibition [20,24,26,27] but it also has to be considered that the upregulation of other TAM receptors may again induce resistance [30]. In nasopharyngeal carcinoma (NPC), Axl expression correlated with distant metastasis and high TNM stage, suggesting Axl as a potential marker for migration and invasion in NPC [31]. However, there is a significant lack of clinical data in HNSCC patients receiving standard-of-care curative treatment that would confirm a prominent prognostic role of Axl expression. For this reason, we tested the prognostic value of the expression levels of Axl and its ligand Gas6 in a retrospective single center cohort in a tissue microarray format.

## 2. Materials and Methods

### 2.1. Patient Material

Survival and clinicopathological data from patients, who had been diagnosed with HNSCC and treated with curative intent at the University Medical Center Hamburg-Eppendorf from 1992–2007 and from 2008–2013, were analyzed retrospectively. Tissue samples were obtained from primary tumors during diagnostic biopsies or surgical resection. The use of archived diagnostic leftover tissues and their analysis for research purposes, as well as patient data analysis, have been approved by local laws (HmbKHG, §12.1) and by the local ethics committee (Ethics commission Hamburg, WF-049/09). The study has been carried out in compliance with the Helsinki Declaration.

### 2.2. Tissue Microarray Construction

Tissue samples were fixed in buffered 4% formalin, embedded in paraffin, and used for tissue microarray construction as previously described [32,33]. Hematoxylin–eosin stained sections were made from each selected primary tumor block to identify representative tumor regions. One tissue cylinder (0.6 mm in diameter) was punched from each tumor using a homemade semi-automated tissue arrayer. For immunohistochemical staining, three-micrometer microarray sections were prepared using the Paraffin Sectioning Aid System (Instrumentics, Hackensack, NJ, USA). Separate tissue microarrays were established from tumors treated between 1992–2007 and 2008–2013 with 95% of tumors diagnosed and treated between 2000 and 2013. Staining and analysis was performed in parallel.

### 2.3. Immunohistochemistry (IHC)

For IHC analyses, freshly cut 3 μm thick tissue microarray sections were analyzed. Immunohistochemistry was performed with an automated stainer (Benchmark XT, Ventana Medical Systems, Oro Valley, AZ, USA), using standard protocols (anti-Gas6 polyclonal goat antibody, R&D Biosystems, # AB 885, pre-treatment 1 hr in citrate buffer cc2, Roche, antibody dilution 1:10; anti-Axl polyclonal goat antibody, R&D Biosystems, # AF 154, pre-treatment 1 hr in citrate buffer cc1, Roche, antibody dilution 1:100). Bound antibodies were detected by the peroxidase method using diaminobenzidine as chromogen (Ventana Medical Systems, # 760-500). All slides were counterstained with alum–hematoxylin. Individual tumor spots were scored based on the staining intensity (0, 1, 2, 3—referring to absent, low, intermediate, or high intensity) of the tumor cells and the fraction of tumor cells stained. The final score for Axl and Gas6 was derived from these two parameters as follows: negative scores had a staining intensity of 0; weak scores had a staining intensity of 1+ in ≤70% of tumor cells or a staining intensity of 2+ in ≤30% of tumor cells; moderate scores had a staining intensity of 1+ in >70% of tumor cells, a staining intensity of 2+ in >30% and ≤70% of tumor cells, or a staining intensity of 3 in ≤30% of tumor cells; strong scores had a staining intensity of 2+ in >70% of tumor cells or a staining intensity of 3 in >30% of tumor cells. The p16 status was determined for oropharyngeal tumors. Spots were scored as positive when ≥70% of tumor cells showed an intermediate or high staining intensity (monoclonal mouse anti-p16^INK4a^ antibody, Nordic-MUbio, clone DCS-50.1/A7: dilution 1:1350).

### 2.4. Analysis of Patient Survival

R (version 3.6.3) and Bioconductor environment [34] were used for data processing, analysis, and evaluation. Survival analyses were performed according to the Kaplan-Meier method and the Log-rank test. Multivariable analyses were performed fitting a Cox proportional hazards regression model (R-packages: *survival* and *survminer* [35,36]). Potential associations between variables were tested using the Pearson correlation coefficient (R-packages: *reshape* and *corrplot* [37,38]). All statistical analyses are to be considered exploratory. The reported *p*-values are two-sided and used as descriptive measures only.

### 2.5. Further Data Analyses

Depiction of protein expression scores were performed using GraphPad Prism 6.

## 3. Results

We assessed the protein expression level of the TAM receptor Axl and its ligand Gas6 in primary HNSCC tumor samples derived from a single center retrospective cohort of patients treated for HNSCC with curative intent in a tissue microarray format. Interpretable stainings were obtained in 362 individual samples. For oropharyngeal tumors (OPSCC), p16-status as an indicator for either HPV-induced or HPV-independent tumorigenesis was available for 158 of 170 samples. The clinicopathological characteristics of the patients with interpretable Axl and/or Gas6 stained samples are presented in Table 1. A majority of 292 patients (80.7%) were treated primarily by surgery, of which 181 (50%) patients received adjuvant RT/RCT and 111 received surgical treatment alone (30.7%). Fifty-nine patients were primarily treated with RT/RCT (16.3%).

### 3.1. Expression of Axl and Gas6 in HNSCC Subsites

**Figure 1:**
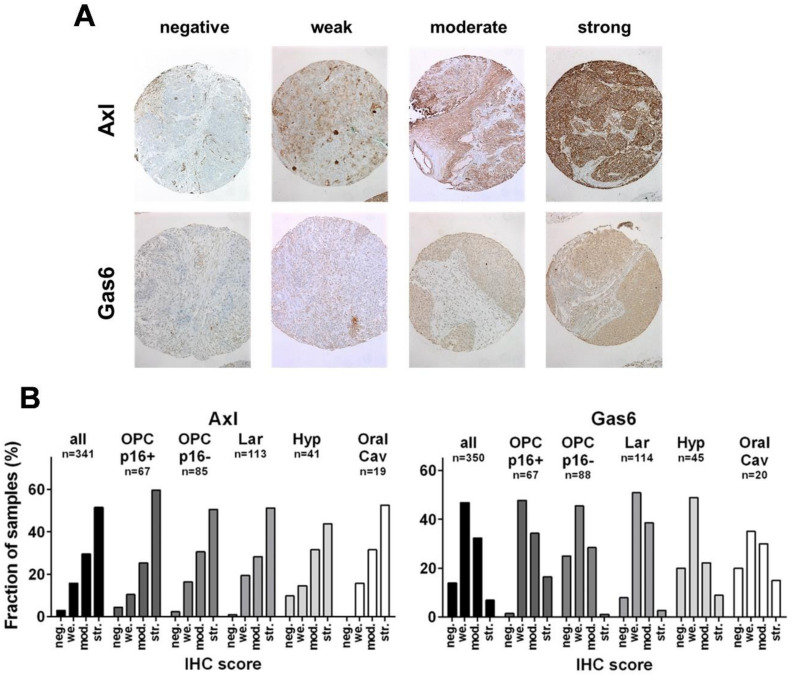
**Immunohistochemical staining.** (**A**) Representative examples of expression score categories. (**B**) Distribution of expression scores in the whole HNSCC population and specific subtypes. neg.—negative, we.—weak, mod.—moderate, str.—strong, IHC—immunohistochemistry.

In a semiquantitative analysis, the immunohistochemical staining of Axl and Gas6 were scored as *absent*, *weak*, *moderate,* or *strong* following a well-established expression score, depending on staining intensity and the fraction of cells stained [39,40] (Figure 1A). For Axl, we observed a highly similar distribution of protein expression across all HNSCC subsites, with the majority of samples showing strong expression (staining intensity 2+ in >70% or 3 in >30% of tumor cells), while an absence of staining was observed in only a minimal fraction of tumors (Figure 1B). For Gas6, we generally observed less intense staining as compared to Axl, and tumors with highest staining intensity (3) were generally not observed. Therefore, all tumors classified as *strong* demonstrated intermediate staining intensity (2) in the majority (>70%) of tumor cells. Most tumors demonstrated weak expression, and those classified as strong were the smallest fraction in all subsites except for p16-positive OPSCC. In these, the fraction of strongly expressing tumors, albeit still being below 20%, was most frequent among the subsites, while unstained tumors were almost completely lacking, which may suggest dependence of these tumors on at least weak Gas6 expression. While Axl staining was largely restricted to the plasma membrane, Gas6 mostly demonstrated an even cytoplasmic staining pattern and some nuclear staining in a subfraction of cases (Figure 1A).

With the data gathered, we performed correlation analyses of the staining intensities of both proteins and T + N-stage as the most important clinical predictors of outcome. Due to the different etiology of HPV-induced tumors, analysis was performed separately in p16-positive OPSCC and a pooled cohort containing p16-negative OPSCC and all non-oropharyngeal tumors (except for the only 5 NPC samples), in which HPV-driven malignancies are reported to represent only a small minority of less than 5% when critically assessed through detection of HPV E6/E7 mRNA in addition to p16 or HPV DNA [41,42,43,44,45,46]. Subsequently, this pooled cohort is referred to as HPV-negative HNSCC. No correlation between Axl or Gas6 expression and T- or N-stage, or between the expression levels of both proteins, was observed in either group, despite their close functional interaction as receptor and ligand (Supplementary Figure S1).

### 3.2. Impact of Axl Expression on Patient Survival

**Figure 2:**
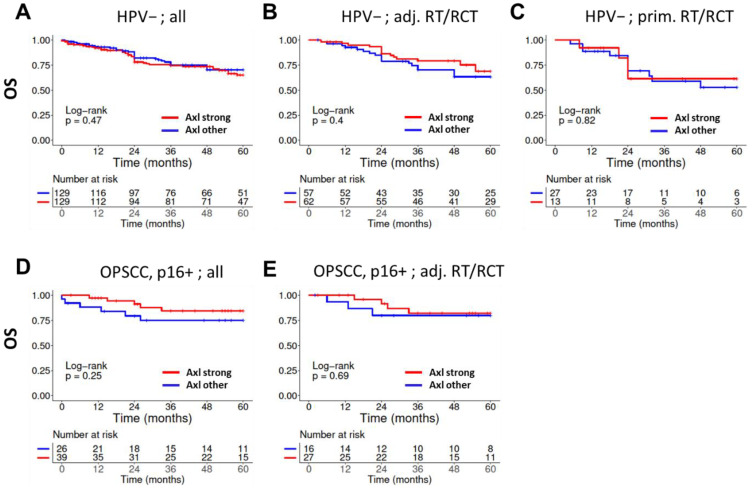
**Association of overall survival with Axl expression and treatment.** Axl expression was categorized as either *strong* or *other*, which includes all samples scored as *negative*, *weak,* or *moderate*. (**A**) All patients with HPV-negative HNSCC. (**B**) Patients with HPV-negative HNSCC treated by surgery and adjuvant RT/RCT and (**C**) with primary RT/RCT. (**D**) All patients with p16-positive OPSCC and (**E**) those treated with surgery and adjuvant RT/RCT.

Due to the difference in tumor biology and prognosis between HPV-associated and independent HNSCC, we performed survival analysis separately in p16-positive OPSCC and the above described HPV-negative HNSCC cohort comprising multiple subsites. Regarding patient outcome, we did not observe a significant effect of Axl expression on overall or recurrence-free survival (OS, RFS) in both cohorts when comparing the large group of patients with *strong* expression score to all others (Figure 2A,D, Supplementary Figure S2A,D). When stratifying for treatment, no significant differences regarding OS or RFS was observed for patient groups treated by surgery plus adjuvant radiotherapy or chemoradiation (RT/RCT) (Figure 2B,E, Supplementary Figure S2B,E) or for patients with HPV-negative tumors treated with primary RT/RCT (Figure 2C, Supplementary Figure S2C). For the subgroup of p16-positive OPSCC, the low number of patients treated primarily with RT/RCT prevented meaningful analyses.

**Figure 3:**
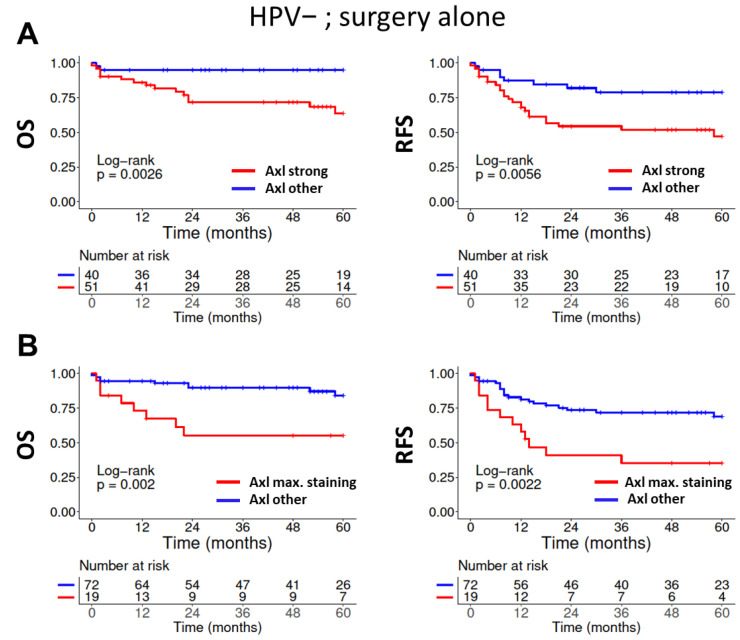
**Association of patient survival and Axl expression in patients with HPV-negative tumors solely treated by surgery.** (**A**) Axl-expression was categorized by the semiquantitative expression score as either *strong* or *other*. (**B**) Axl expression was categorized by staining intensity. Only tumors showing the highest intensity (3) in more than 30% of tumor cells were categorized to the *maximum (max.) staining* group, which defines a smaller population than as compared to the *strong* expression score.

In contrast to the results observed for surgery plus adjuvant RT/RCT, a clear association of Axl expression and patient outcome was evident in patients with HPV-negative HNSCC treated by surgery alone. We observed statistically significant inferior OS (*p* = 0.0026) and RFS (*p* = 0.0056) in patients with *strong* Axl expression (Figure 3A). Stratified by individual subsites, trends towards inferior outcome correlating with *strong* expression were evident, but patient numbers were mostly insufficient to draw solid conclusions. A significantly inferior OS was found in p16-negative OPSCC (*p* = 0.018) (Supplementary Figure S3). Since our semiquantitative expression score classified about half of tumors as *strong* by including a large fraction of samples with intermediate staining intensity in a majority of tumor cells, we performed an additional analysis specifically focusing on tumors with the highest expression levels (staining intensity 3) in more than 30% of tumor cells. We obtained a comparably small subgroup with an especially bad prognosis under sole surgical treatment (20.9%; OS: *p* = 0.002; RFS: *p* = 0.0022), which might particularly benefit from adjuvant RT/RCT (Figure 3B). Finally, we analyzed patients treated by surgery alone as two separate, independent groups treated between (i) 1992–2007 and (ii) 2008–2013, corresponding to individual TMA establishment. Significantly inferior OS for those with *strong* Axl expression was consistently observed in both cohorts (*p* = 0.031 and *p* = 0.035, respectively). RFS was significantly inferior in the former (*p* = 0.0059) and trended towards inferiority in the latter cohort (*p* = 0.11) (Supplementary Figure S4). Unlike the situation in HPV-negative HNSCC, no tendencies towards inferior survival with strong Axl expression were observed in p16-positive OPSCC treated by surgery alone. In fact, we even observed a trend towards favorable OS (*p* = 0.07) and significantly better RFS (*p* = 0.048). However, due to the frequency of lymph node metastases in this group, the number of patients treated with surgery alone was low (n = 11). Therefore, interpretation is limited and can only be considered for hypothesis generation (Supplementary Figure S5).

### 3.3. Impact of Gas6 Expression on Patient Survival

**Figure 4:**
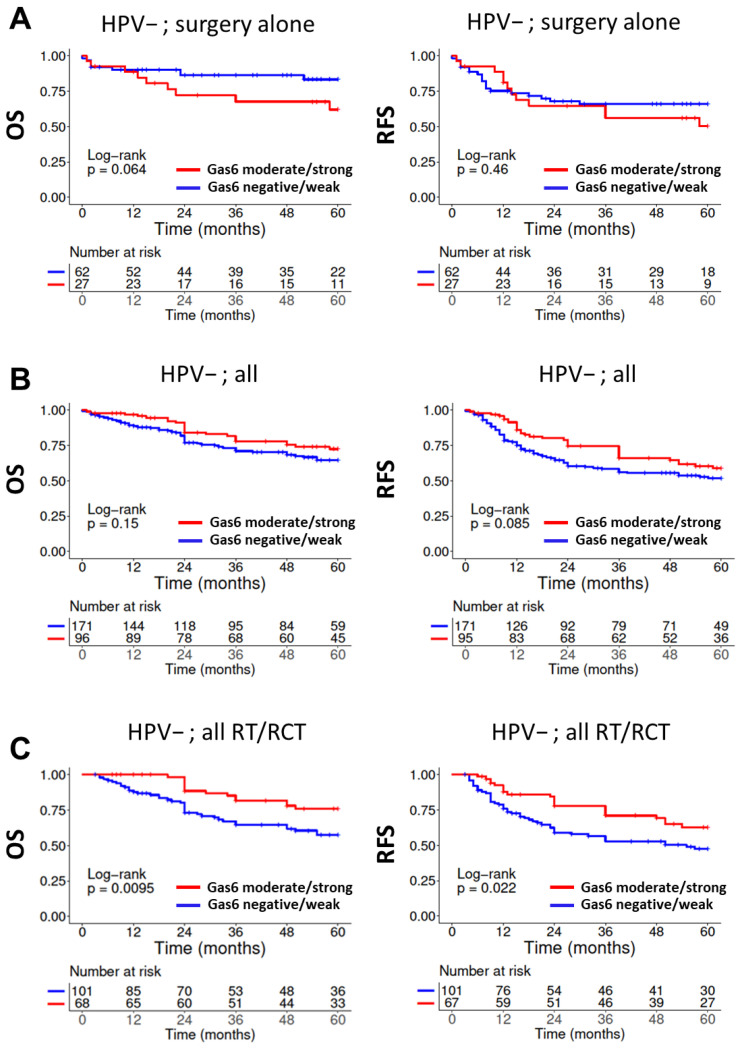
**Association of patient survival and Gas6 expression in patients with HPV-negative tumors.** Patients were separated into a lower *(negative/weak)* and higher *(moderate/strong)* expression group. (**A**) OS and RFS of patients treated by surgery alone. (**B**) OS and RFS of patients irrespective of treatment. (**C**) OS and RFS of patients treated by RT in any form.

Since Gas6 staining was far less intense than Axl staining and a weak expression score was the most abundant (Figure 1B), we separated our cohort by tumors, categorized as “absent/weak” and “moderate/strong”. Similar to Axl, we observed a trend towards inferior OS upon higher Gas6 expression in patients with HPV-negative HNSCC treated by surgery alone. Differing from Axl, results were not significant, and no trend was observed for RFS, making any judgment toward prognosis difficult (Figure 4A). While we did not detect a significant association for the whole HPV-negative cohort (Figure 4B) or HPV-negative patients treated with surgery plus adjuvant RT/RCT or primary RT/RCT (Supplementary Figure S6), we observed trends towards superior survival in the group with higher expression. Indeed, significance was reached for a pooled cohort including all HPV-negative HNSCC treated with RT in any form (adj. RT/RCT + primary RT/RCT) for OS and RFS (OS: *p* = 0.0095; RFS: *p* = 0.022) (Figure 4C). For p16-positive OPSCC, no associations were observed between Gas6 expression and survival, regardless of treatment (Supplementary Figure S7).

### 3.4. Multivariable Analyses

In multivariable analyses including expression score, T- and N-stage, sex, and age, strong Axl expression was a potent independent predictor of OS and RFS in patients with HPV-negative tumors treated by surgery alone (OS: HR 10.03, *p* = 0.0036; RFS HR 3.06, *p* = 0.0080). T-stage (OS and RFS) and age (OS only) were also significant. For HPV-negative patients treated by radiotherapy in any form, high Gas6 expression proved to be an independent predictor of superior survival (OS: HR 0.34, *p* = 0.0006; RFS: HR 0.46, *p* = 0.0027). N-stage (OS and RFS) and age (OS only) were also significantly associated with inferior survival (Table 2).

## 4. Discussion

Our TMA analysis of a retrospective HNSCC cohort suggests that strong expression of Axl is a major and independent prognostic factor associated with inferior outcome after sole surgical treatment in patients with HPV-negative tumors (Figure 3, Table 2). Since we observed no inferior outcome in patients that received adjuvant or primary RT/RCT, we propose that Axl should be conclusively tested for its capability as a predictive biomarker for the addition of RT/RCT for patients with strong Axl expression that might otherwise be treated with surgery alone. For this purpose, and when focusing on tumors with the highest staining intensity, Axl expression may define a comparably small subgroup with clearly inferior prognosis (Figure 3B). Based on the available preclinical data regarding Axl in HNSCC, these results were somewhat unexpected. Axl was repeatedly described as a resistance factor, especially against EGFR or PI3K inhibition [20,26,27], which cannot be addressed with our cohort. Axl was also described as a resistance factor against DNA damaging therapies, such as PARP inhibition or radio- and chemo-therapy, as inhibition of Axl dampens the cellular DNA damage response [20,26,27,29,47]. However, our TMA analysis does not support a role for the Axl expression level as a major determinant of radio-resistance in HNSCC. While we observed inferior survival after surgery alone to be associated with strong Axl expression in HPV-negative tumors, patients treated by surgery plus adjuvant RT/RCT demonstrated a trend towards favorable RFS (*p* = 0.072) (Supplementary Figure S2B).

Possible explanations for the inferior outcome after surgery alone could be an enhanced migration of tumor cells associated with Axl expression, thus, single tumor cells or tiny patches may reside outside the resection margin, promoting local and locoregional recurrence. Also, as Axl was repeatedly reported to be a driver for epithelial-to-mesenchymal transition (EMT) [48], strong expression of Axl may also drive the formation of distant metastasis in HNSCC, although in this case, an impact of local RT is more difficult to assume. Indeed, preclinical studies hint to enhanced migration and invasion associated with Axl in HNSCC [20,25].

Similar to Axl, its ligand Gas6 was also not associated with worse outcomes after RT/RCT and in fact, we even observed significantly better OS and RFS in the higher expressing subgroups (Figure 4C, Table 2). Additional retrospective analyses and—at best—prospective studies will be necessary to further clarify the impact of both proteins on patient outcome in HNSCC. Mechanistic studies are required to clarify a potential influence of Gas6 on radio- and chemo-sensitivity.

Our study has several limitations. The data represent a retrospective cohort analysis, and the specimens were collected over a considerable time frame (95% of tumors treated between 2000 and 2013), with evolving techniques in both surgery and radiotherapy occurring throughout. Our cohort also lacks HPV DNA or RNA status, relying on p16 in OPSCC as a surrogate marker for HPV-induced tumorigenesis. However, in OPSCC p16 is well established [49], and in non-oropharyngeal subsites, recent, thorough analyses of the HPV status has clearly demonstrated that active HPV infections are very rarely the cause of tumorigenesis [41,42,43,44,45,46]. We therefore think that although we cannot exclude the possibility that single HPV-positive tumors are included in our pooled cohort, as well as single HPV-negative OPSCC despite p16 staining, they are highly unlikely to substantially skew the results.

In summary, we propose that Axl may constitute a predictive marker indicating the need for (adjuvant) RT/RCT in the treatment of HPV-negative HNSCC, and Gas6 may even constitute a positive prognostic marker in HPV-negative HNSCC treated with RT/RCT-containing regimes. Further investigations on the clinical impact of Axl and Gas6 expression are necessary to clarify the, to some extent, contradictory results of our retrospective observations and previous, mostly mechanistic studies.

## Figures and Tables

**Table 1:** **Clinicopathological characteristics of patient samples.** T- and N-classification were performed according the 7th edition of the Union for International Cancer Control (UICC) and represents pathological staging for resected tumors and clinical staging for those treated with definitive RT/RCT. For 12 spots of oropharyngeal tumors, p16 status was not available (n.a.) because of indistinct scoring (intermediate or high staining intensity in ≥40 but ˂70% of tumor cells) or because of a lack of tumor tissue in the p16-stained tissue microarray section.

Patient Characteristics		
**Interpretable staining, number (%)**		
	Axl and/or Gas6	362 (100)
	Axl	341 (94.2)
	Gas6	350 (96.7)
**Age**, median (range)		60 (32–85)
**Sex**, number (%)		
	male	281 (77.6)
	female	81 (22.4)
**Location**, number (%)		
	Oropharynx	170 (47)
	p16+ (% of OPSCC)	68 (40)
	p16− (% of OPSCC)	90 (52.9)
	p16 n.a. (% of OPSCC)	12 (7.1)
	Larynx	120 (33.1)
	Hypopharynx	46 (12.7)
	Oral cavity	21 (5.8)
	Nasopharynx	5 (1.4)
**T classification**, number (%)		
	T1	83 (22.9)
	T2	106 (29.3)
	T3	88 (24.3)
	T4	84 (23.2)
	n.a.	1 (0.3)
**N classification**, number (%)		
	N0	159 (43.9)
	N1	53 (14.6)
	N2	131 (36.2)
	N3	19 (5.3)
**Therapy**, number (%)		
	surgery	111 (30.7)
	surgery + (chemo)radiation	181 (50)
	chemoradiation	50 (13.8)
	radiotherapy	9 (2.5)
	other	5 (1.4)
	n.a.	6 (1.7)

**Table 2:** **Multivariable analysis.** Asterisks indicate significant associations of variables and survival, with *, ** and *** indicating *p* < 0.05, *p* < 0.01, and *p* < 0.001, respectively (Cox proportional hazards regression). The table includes all tested variables of the respective analyses. Correlation analyses of the variables did not indicate meaningful associations in the respected subgroups, except for a weak association between T- and N-stage (not shown).

Variables		Overall Survival	
	HR	95% CI	*p*-value
**HPV-, surgery only**			
Axl	10.035	2.13–47.26	** 0.0036
T-stage	1.661	1.011–2.728	* 0.0454
N-stage	1.339	0.762–2.355	0.3105
age	2.088	1.161–3.755	* 0.0139
sex (m,f)	0.380	0.0848–1.704	0.2063
**HPV-, RT in any form**			
Gas6	0.340	0.184–0.628	*** 0.0006
T-stage	1.269	0.962–1.676	0.0923
N-stage	1.598	1.190–2.145	** 0.0018
age	1.512	1.131–2.022	** 0.0053
sex (m,f)	0.880	0.467-1.656	0.6913
**Variables**		**Recurrence free survival**	
	**HR**	**95% CI**	***p*** **-value**
**HPV-, surgery only**			
Axl	3.061	1.338–7.002	** 0.0080
T-stage	1.722	1.206–2.458	** 0.0028
N-stage	1.212	0.7982–1.840	0.3670
age	1.003	0.6694–1.502	0.9895
sex (m,f)	1.034	0.4228–2.530	0.9410
**HPV-, RT in any form**			
Gas6	0.459	0.276–0.763	** 0.0027
T-stage	1.198	0.946–1.516	0.1337
N-stage	1.438	1.229–1.840	** 0.0040
age	1.233	0.965–1.576	0.0947
sex (m,f)	1.136	0.668–1.931	0.6380

## Data Availability

The data presented in this study are available on reasonable request from the corresponding authors. The data are not publicly available due to data protection issues.

## Supplementary Materials

The following supporting information can be downloaded at: https://www.mdpi.com/article/10.3390/cancers14071829/s1.
