# Supplementary material for: Tissue Microarray Analyses Suggest Axl as a Predictive Biomarker in HPV-Negative Head and Neck Cancer
Source: Cancers (Basel). 2022 Apr 5;14(7):1829. doi: 10.3390/cancers14071829 (PMC8997923; doi:10.3390/cancers14071829)
Supplement: Supplementary file 1 [file cancers-14-01829-s001.zip › cancers-1635873-SI.pdf]

cancers

Supplementary Figures

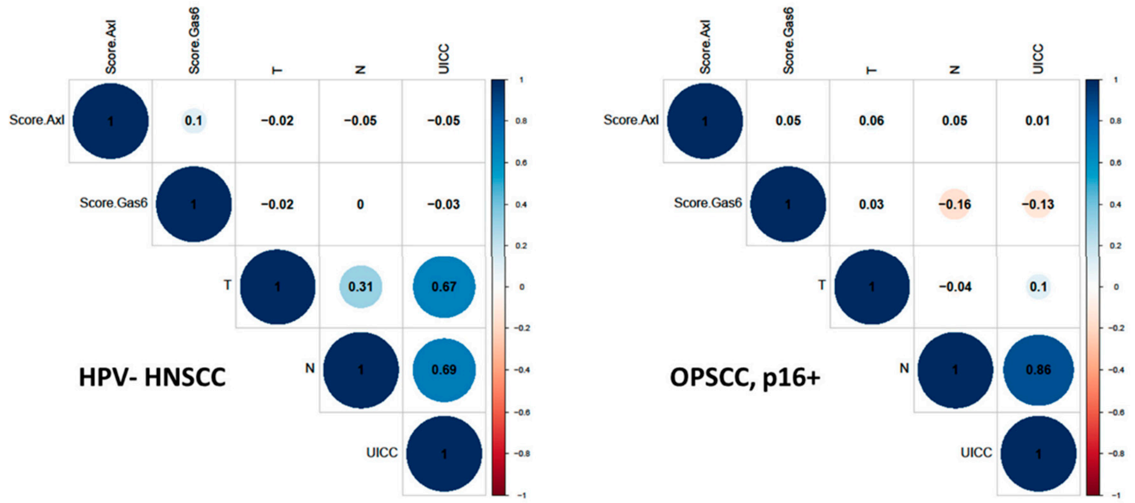

Supplementary Figure S1. Correlation analyses. Correlation analyses of Axl and Gas6 expression scores with each other and with T-, N- and UICC-stage (7th edition). As expected, T and N-stage showa clear correlation with UICC-stage in HPV-negative HNSCC and N-stage with UICC-stage in p16+ OPSCC. Axl and Gas6 do not demonstrate a meaningful correlation with any parameter with each otheror any of the clinicopathological parameters.

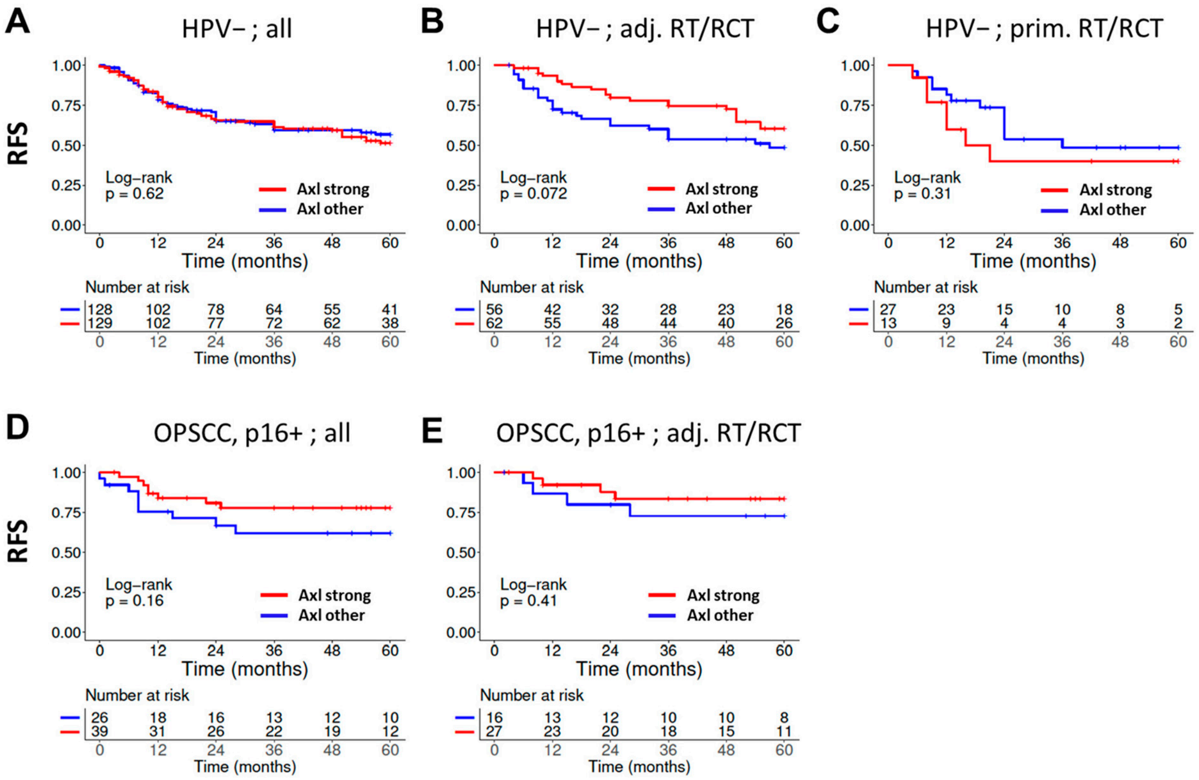

Supplementary Figure S2. Association of recurrence free survival with Axl expression and treatment. Axl expression was categorized as either *strong* or *other*, which includes all samples scored as *negative*, *weak*, or *moderate*. (**A**) All patients with HPV-negative HNSCC. (**B**) Patients with HPV-

negative HNSCC treated by surgery and adjuvant RT/RCT and (**C**) with primary RT/RCT. (**D**) All patients with p16-positive OPSCC and (**E**) those treated with surgery and adjuvant RT/RCT.

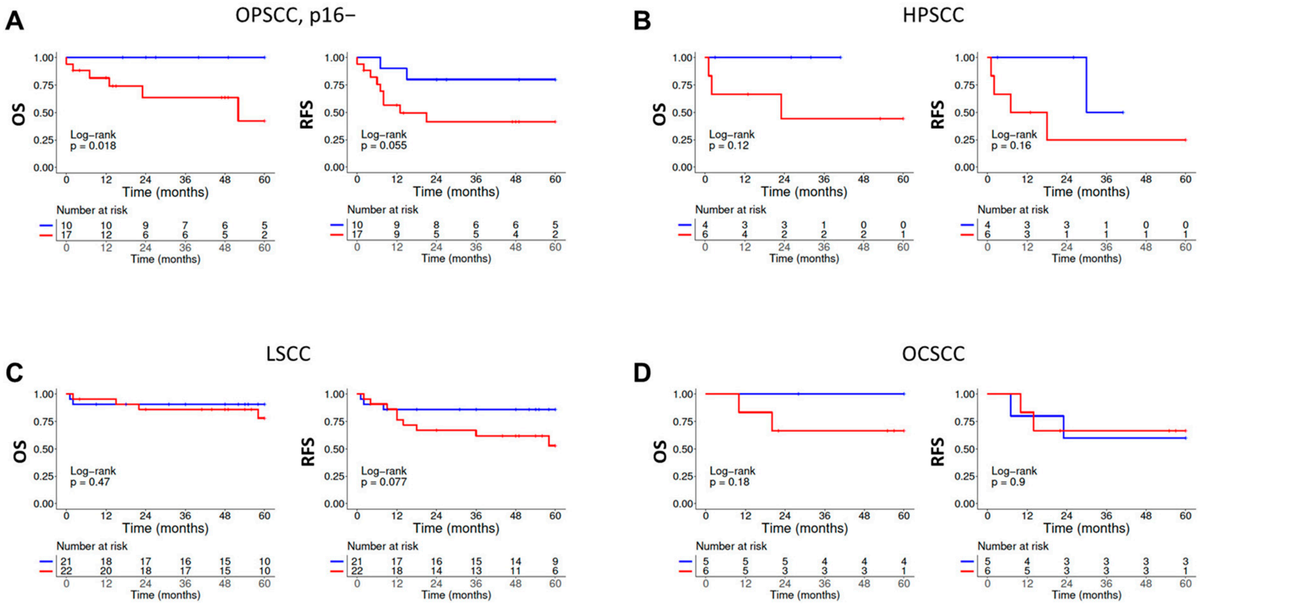

Supplementary Figure S3. Association of patient survival and Axl staining after surgery alone in HNSCC sublocations. Patient survival in dependence of Axl-expression as categorized by the semiquantitative expression score as either *strong* or *other*. (**A**) Survival of patients with p16-negative OPSCC. (**B**) Survival of patients with hypopharyngeal tumors (HSCC). (**C**) Survival of patients with laryngeal tumors (LSCC). (**D**) Survival of patients with oral cavity tumors (OCSCC).

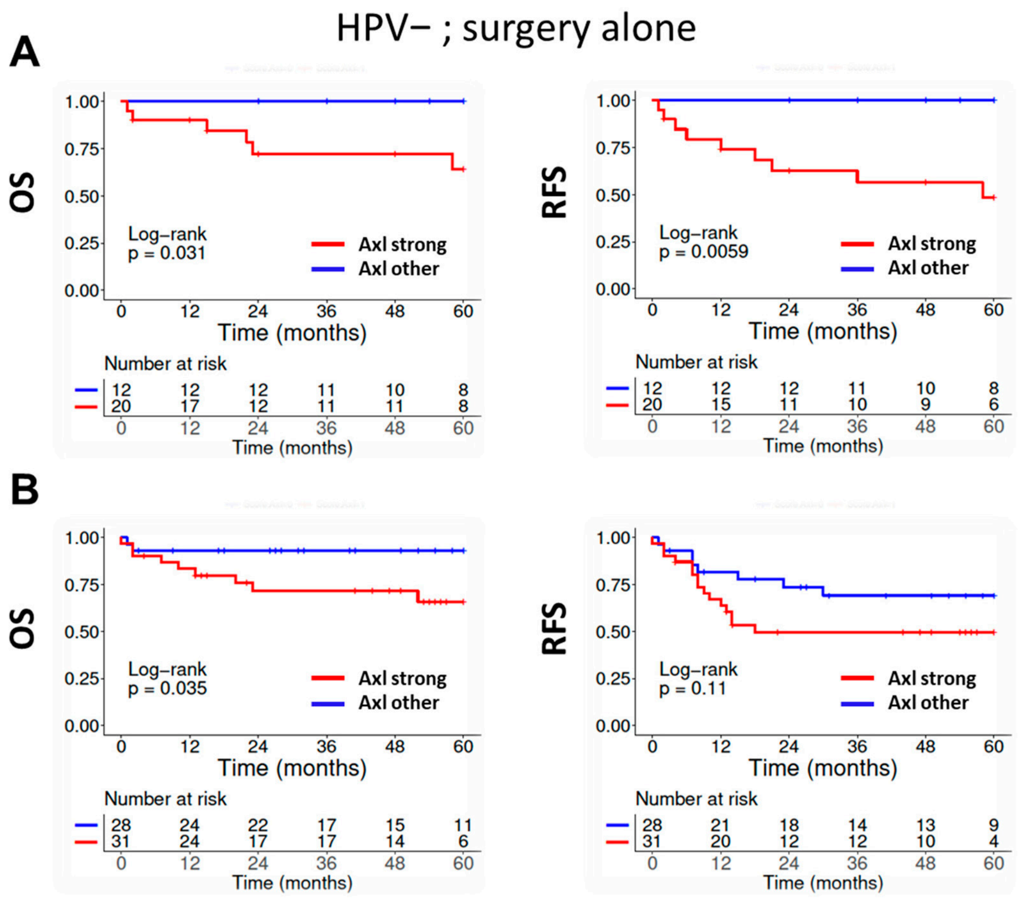

Supplementary Figure S4. Association of survival and Axl expression in patients treated by surgery alone in the (A) earlier (treated between 1992-2007) and (B) later (treated between 2008-2013) patient cohort. Patient survival in dependence of Axl-expression as categorized by the semiquantitative expression score as either *strong* or *other*.

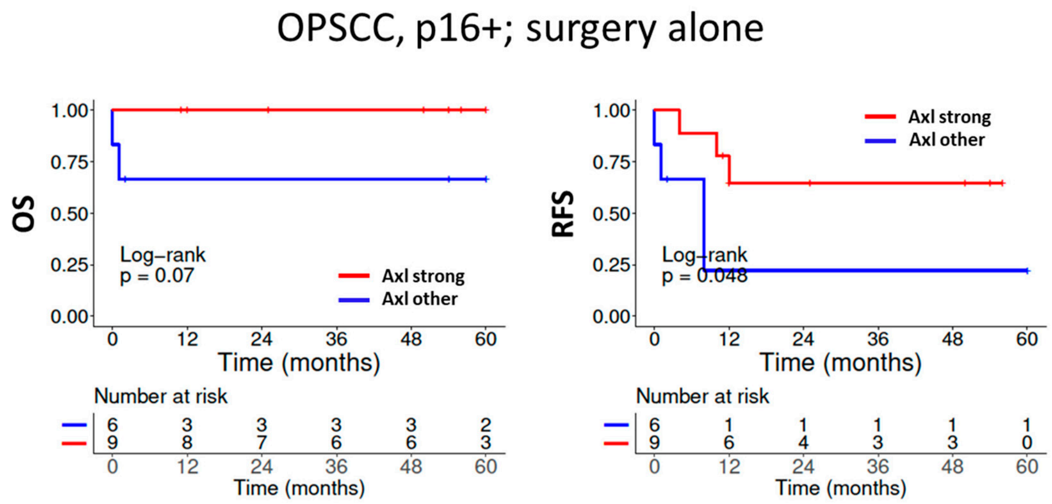

Supplementary Figure S5. Survival in relation to Axl expression in patients with p16-positive OPSCC treated by surgery alone. Patient survival in dependence of Axl-expression as categorized bythe semiquantitative expression score as either *strong* or *other*.

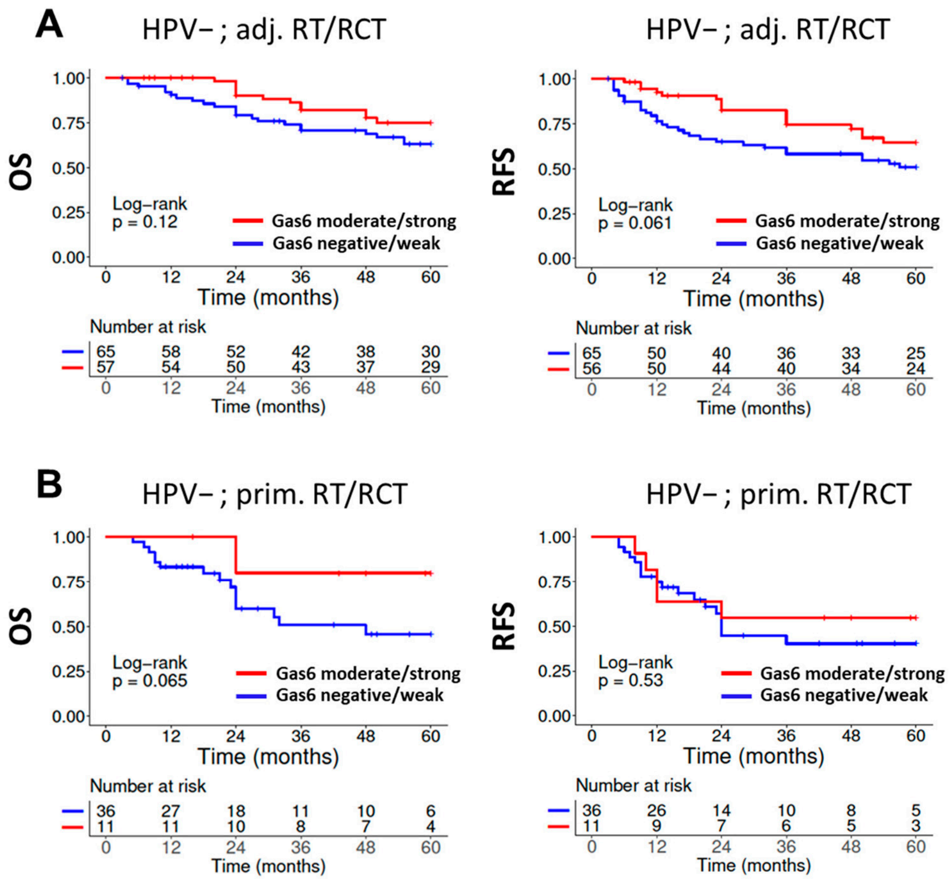

Supplementary Figure S6: Survival in relation to Gas6 expression in patients with HPV-negative HNSCC treated by adjuvant or primary radio(chemo)therapy. Patient survival in dependence of Gas6-expression was categorized by the semiquantitative expression score as either *negative/weak* or *moderate/strong*. (**A**) Treatment by surgery plus adjuvant RT/RCT. (**B**) Treatment by primary RT/RCT.

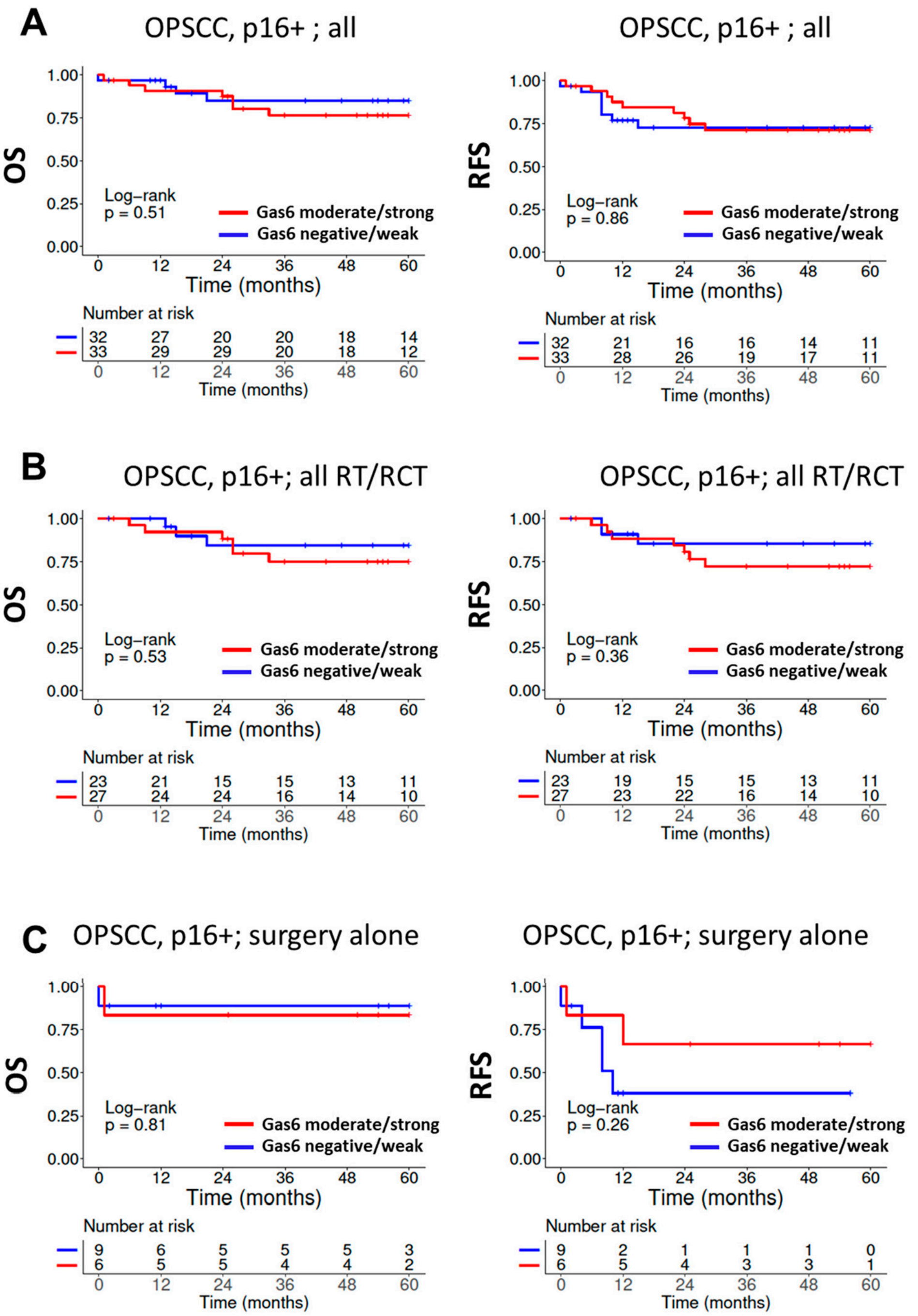

Supplementary Figure S7: Survival of patients with p16-positive OPSCC in dependence of Gas6 expression and treatment. Patient survival in dependence of Gas6-expression was categorized by the semiquantitative expression score as either *negative/weak* or *moderate/strong*. (**A**) All patients with p16-positive OPSCC. (**B**) Patients treated by RT in any form. (**C**) Patients treated by surgery alone.
